# Supplementary material for: Durability of Immune Response to ChAdOx1-nCoV-19 Vaccine in Solid Cancer Patients Undergoing Anticancer Treatment
Source: Vaccines (Basel). 2022 Oct 5;10(10):1662. doi: 10.3390/vaccines10101662 (PMC9609471; doi:10.3390/vaccines10101662)
Supplement: Supplementary file 1 [file vaccines-10-01662-s001.zip › vaccines-1893643-supplementary.pdf]

Figure S1. Consort diagram

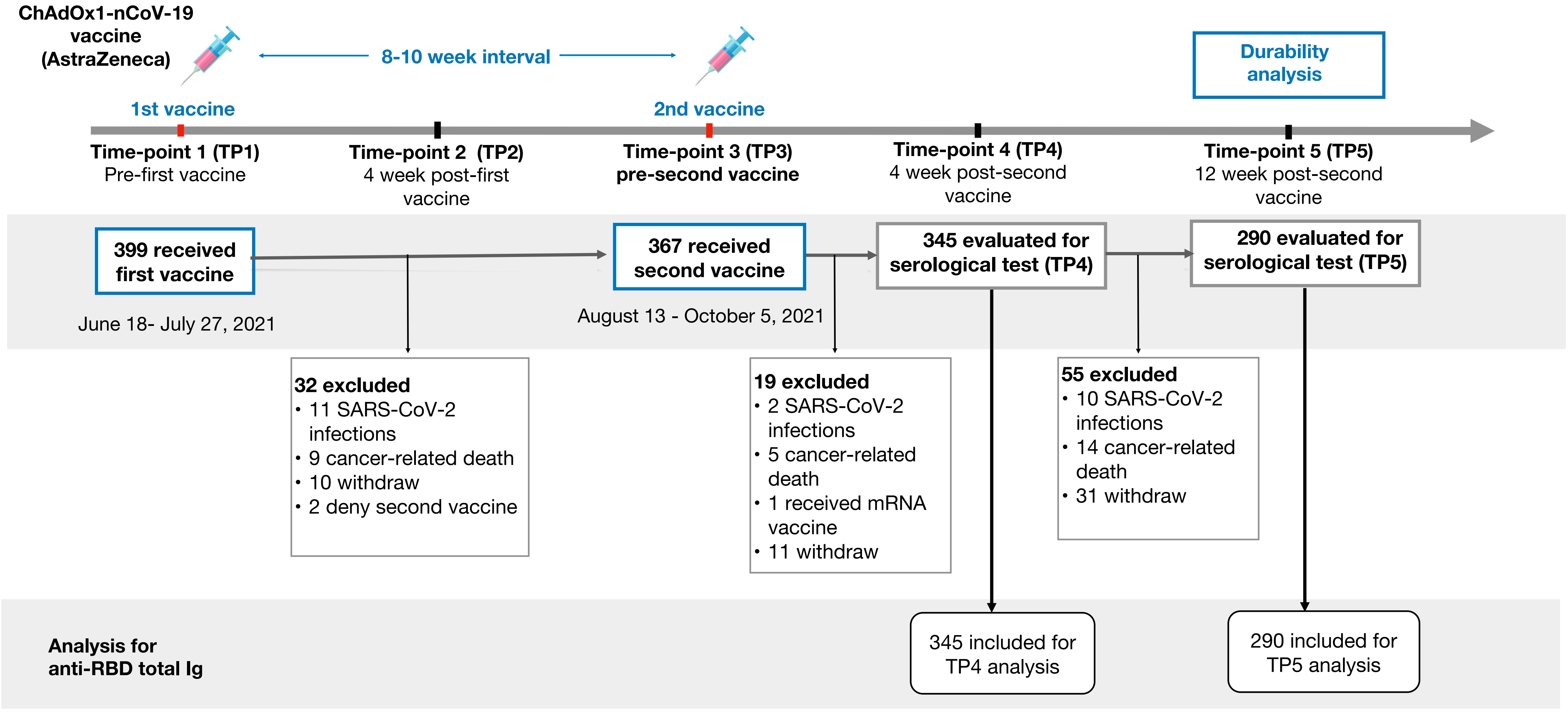

Figure S2. Proportion of late titer rising among cancer patients that received immunotherapy, chemotherapy or TKI compared to healthy individuals

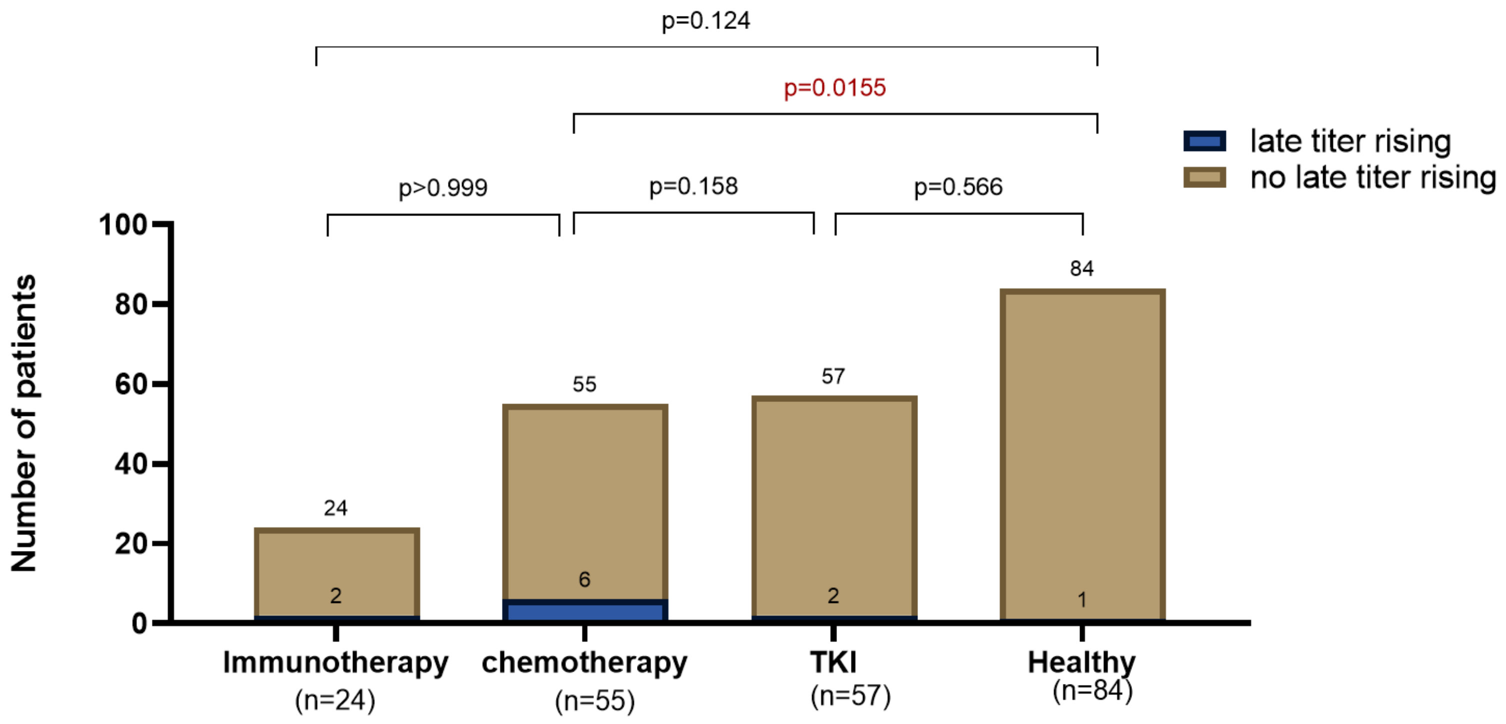

Figure S3. Proportion of inadequate response among cancer patients that received immunotherapy, chemotherapy or TKI compared to healthy individuals

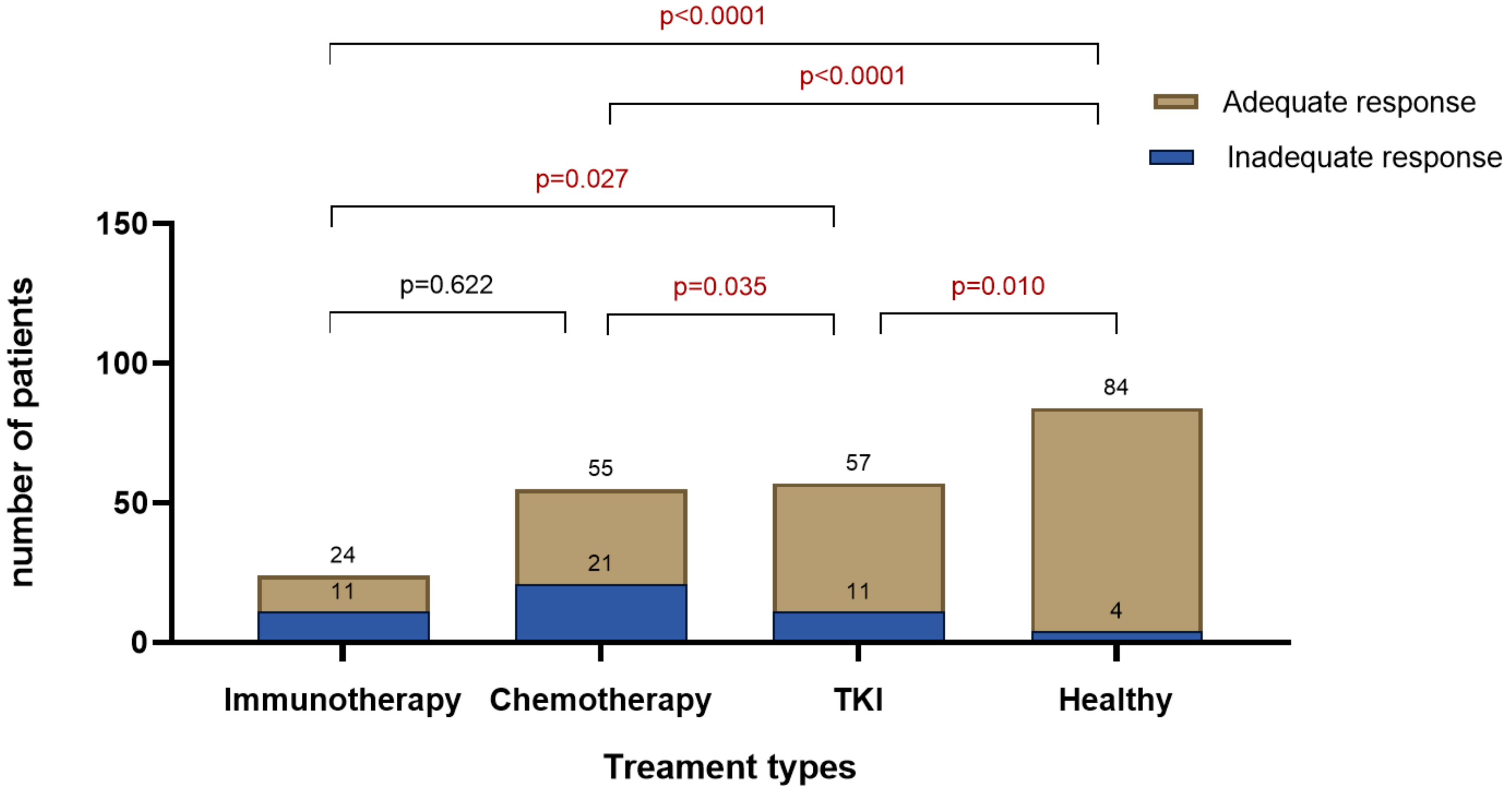

Table S1 Factors associated with adequate response and inadequate response (Using concentration titer cutoff of 210 U/ml by current FDA Emergency use Authorization guidelines defined for high titer for COVID-19 convalescent plasma)

Factors	Concentration titer >210 U/ml "Adequate response" (N=212)	Concentration titer <210 U/ml "Non-adequate response" (N=78)	Univariable logistic model on low titer status		Multivariable logistic model on low titer status	
			Odds ratio (95% CI)	p-value	Odds ratio (95% CI)	p-value
Age (years)						
<60	98 (46)	34 (44)	Reference	Reference		
≥60	114 (54)	44 (56)	1.11 (0.66, 1.88)	0.689		
Sex						
Female	146 (69)	40 (51)	Reference	Reference	Reference	Reference
Male	66 (31)	38 (49)	2.10 (1.24, 3.57)	0.0061	1.38 (0.77, 2.46)	0.280
BMI						
<18.5	26 (12)	10 (13)	1.25 (0.54, 2.91)	0.604		
18.5-22.9	91 (43)	28 (36)	Reference	Reference		
23.0-24.9	35 (17)	19 (24)	1.76 (0.88, 3.56)	0.112		
≥25	60 (28)	21 (27)	1.14 (0.59, 2.19)	0.699		
ECOG						
0-1	202 (95)	72 (92)	Reference	Reference		
2-3	10 (5)	6 (8)	1.68 (0.59, 4.80)	0.330		
Comorbidity						
No	118 (56)	29 (37)	Reference	Reference	Reference	Reference
Yes	94 (44)	49 (63)	2.12 (1.24, 3.62)	0.0057	1.90 (1.08, 3.35)	0.026
Cancer type						
Lung	54 (26)	21 (30)	1.50 (0.74, 3.03)	0.261		
Breast	77 (37)	20 (28)	Reference	Reference		
Colorectal	44 (21)	15 (21)	1.31 (0.61, 2.82)	0.486		
Upper GI malignancy	13 (6)	7 (10)	2.07 (0.73, 5.88)	0.170		
Genitourinary	6 (3)	3 (4)	1.47 (0.57, 3.80)	0.430		
Soft tissue sarcoma and skin cancer	15 (7)	5 (7)				
Treatment type						
CMT	99 (47)	41 (53)	1.64 (0.86, 3.12)	0.132	1.67 (0.85, 3.26)	0.133
Immunotherapy	17 (8)	18 (23)	4.24 (1.81, 9.91)	0.0009	3.23 (1.31, 7.94)	0.011
TKI	57 (27)	15 (19)	Reference	Reference	Reference	Reference
CDK4/6 inhibitor	12 (6)	2 (3)				
Biologic agent	15 (7)	1 (1)	0.30 (0.06, 1.37)	0.120	0.32 (0.07, 1.51)	0.151
Anti-hormonal treatment	12 (6)	1 (1)				

Hx treatment interruption/cessation						
No	166 (78)	61 (78)	Reference	Reference		
Yes	46 (22)	17 (22)	1.00 (0.54, 1.89)	0.986		
Steroid use						
No	111 (52)	33 (42)	Reference	Reference		
Yes	101 (48)	45 (58)	1.50 (0.89, 2.53)	0.130		

Data are presented as n (%).

Table S2 Factors associated with late-rising immune response in oncologic patients

Factors	Normal (N=240)	Late response (N=50)	Univariable logistic analysis		Multivariable logistic analysis	
			Odds ratio (95% CI)	p-value	Odds ratio (95% CI)	p-value
Age (years)						
<60	107 (45)	25 (50)	Reference	Reference		
≥60	133 (55)	25 (50)	0.80 (0.44, 1.48)	0.485		
Sex						
Female	150 (63)	36 (72)	Reference	Reference		
Male	90 (38)	14 (28)	0.65 (0.33, 1.27)	0.205		
BMI						
<18.5	32 (13)	4 (8)	0.49 (0.16, 1.53)	0.223		
18.5-22.9	95 (40)	24 (48)	Reference	Reference		
23.0-24.9	47 (20)	7 (14)	0.59 (0.24, 1.47)	0.256		
≥25	66 (28)	15 (30)	0.90 (0.44, 1.84)	0.773		
ECOG						
0-1	228 (95)	46 (92)	Reference	Reference		
2-3	12 (5)	4 (8)	1.65 (0.51, 5.35)	0.402		
Comorbidity						
No	119 (50)	28 (56)	Reference	Reference		
Yes	121 (50)	22 (44)	0.77 (0.42, 1.43)	0.410		
Cancer type						
Breast	72 (31)	25 (56)	3.99 (1.54, 10.33)	0.0043	1.94 (0.58, 6.53)	0.284
Lung	69 (29)	6 (13)	Reference	Reference	Reference	Reference
Colorectal	49 (21)	10 (22)	2.35 (0.80, 6.89)	0.120	0.84 (0.22, 3.26)	0.801
Upper GI malignancy	17 (7)	3 (7)	2.03 (0.46, 8.95)	0.350	1.17 (0.23, 5.89)	0.847
Genitourinary	8 (3)	1 (2)	0.41 (0.05, 3.57)	0.420	0.35 (0.04, 3.11)	0.346
Soft tissue sarcoma and skin cancer	20 (9)	0 (0)				
Treatment type						
CMT	105 (44)	36 (72)	4.51 (1.81, 11.24)	0.0012	4.31 (1.10, 16.96)	0.036
Immunotherapy	33 (14)	2 (4)	0.80 (0.15, 4.16)	0.789	1.02 (0.18, 5.69)	0.982
TKI	68 (28)	3 (6)	Reference	Reference	Reference	Reference
CDK4/6 inhibitor	11 (5)	3 (6)				
Biologic agent	11 (5)	5 (10)	3.43 (1.01, 11.67)	0.048	1.95 (0.46, 8.22)	0.364
Anti-hormonal treatment	12 (5)	1 (2)				
Treatment interruption/cessation						
No	190 (79)	37 (74)	Reference	Reference		
Yes	50 (21)	13 (26)	1.34 (0.66, 2.70)	0.421		
Steroid use						
No	127 (53)	17 (34)	Reference	Reference	Reference	Reference

Yes	113 (47)	33 (66)	2.18 (1.15, 4.13)	0.017	0.74 (0.28, 1.96)	0.559

Data are presented as n (%)
